# The Role of Omega-3 Polyunsaturated Fatty Acids in the Treatment of Patients with Acute Respiratory Distress Syndrome: A Clinical Review

**DOI:** 10.1155/2015/653750

**Published:** 2015-08-03

**Authors:** M. García de Acilu, S. Leal, B. Caralt, O. Roca, J. Sabater, J. R. Masclans

**Affiliations:** ^1^Critical Care Department, Vall d'Hebron University Hospital, Vall d'Hebron Research Institute, 08035 Barcelona, Spain; ^2^Ciber Enfermedades Respiratorias (CIBERES), Instituto de Salud Carlos III, 28029 Madrid, Spain; ^3^Critical Care Department, Bellvitge University Hospital, 08907 Barcelona, Spain

## Abstract

Acute respiratory distress syndrome (ARDS) is defined as the acute onset of noncardiogenic edema and subsequent gas-exchange impairment due to a severe inflammatory process. Recent report on the prognostic value of eicosanoids in patients with ARDS suggests that modulating the inflammatory response through the use of polyunsaturated fatty acids may be a useful strategy for ARDS treatment. The use of enteral diets enriched with eicosapentaenoic acid (EPA) and gamma-linolenic acid (GLA) has reported promising results, showing an improvement in respiratory variables and haemodynamics. However, the interpretation of the studies is limited by their heterogeneity and methodology and the effect of *ω*-3 fatty acid-enriched lipid emulsion or enteral diets on patients with ARDS remains unclear. Therefore, the routine use of *ω*-3 fatty acid-enriched nutrition cannot be recommended and further large, homogeneous, and high-quality clinical trials need to be conducted to clarify the effectiveness of *ω*-3 polyunsaturated fatty acids.

## 1. Introduction

Acute respiratory distress syndrome (ARDS), defined as the acute onset of noncardiogenic edema and subsequent gas-exchange impairment due to a severe inflammatory process, affects 78.9 cases per 100,000 person-years in the US and is one of the main reasons for ICU admission [1]. Despite recent advances in overall support, it is still associated with high rates of mortality [1, 2], reduced quality of life [3, 4], and increased healthcare costs [5].

Adjunctive nutritional support is a strategy that is currently receiving growing attention in the management of critically ill patients. In particular, it has been suggested that supplementation with *ω*-3 fatty acids may modulate the inflammatory response in ARDS, although the evidence compiled so far is limited. The aim of this review is, therefore, to discuss recent evidence regarding the role of *ω*-3 fatty acids-enriched diets in patients with ARDS.

## 2. Acute Respiratory Distress Syndrome

According to the latest review by a panel of experts from the European Society of Intensive Care Medicine, the Society of Critical Care Medicine, and the American Thoracic Society [6] (Table 1), ARDS is defined as the onset of acute respiratory failure (P_a_O_2_/F_I_O_2_ < 300 mmHg, with a minimum level of positive end expiratory pressure) with bilateral opacities within a week of a known clinical insult, not fully explained by cardiac failure or fluid overload (using objective assessment tools such as echocardiography to exclude hydrostatic oedema). The clinical insult may be either intra or extrapulmonary. The most frequent causes of ARDS are pneumonia and extrapulmonary severe sepsis [7–9].

Regardless of the primary insult, the lung response has classically been considered as a stereotypical process, characterized by the activation of inflammatory, coagulation, and fibrinolytic systems that lead to lung inflammation and the subsequent epithelial-endothelial barrier injury [10]. Inflammatory mediators may also cause loss of the vascular tone leading to vasoconstriction and creating parched areas of lung destruction [11].

The inflammatory cascade involves large numbers of inflammatory cells and mediators, many of which are directly produced in the lung. It is accompanied by a change in bronchoalveolar lavage (BAL) cellularity, due to neutrophils migration [12]. Neutrophil migration and activation may, then, be the main trigger of lung injury. They produce toxic molecules, chemokines (e.g., IL-1, IL-8, NTFa, L-selectin, and CXCL-CXCR complex), adhesion molecules (e.g., ICAM, PECAM) [13, 14], and procoagulant substances [15]. It has been proved that during lung injury there are proinflammatory as well as anti-inflammatory mediators in BAL fluid and serum, suggesting that it may be the balance between them that regulates lung damage and repair [13].

ARDS is, therefore, a heterogeneous syndrome with no specific treatment. In addition to addressing the primary insult and providing support measures, many therapeutic options have been tested in order to improve the clinical outcome in these patients. Until now, protective mechanical ventilation [16] and prone-positioning in the more severe patients [17] are the only strategies that have clearly demonstrated their usefulness for ARDS management. Other treatments such as neuromuscular blockage, vasodilators, anti-inflammatory drugs, extracorporeal support, or high frequency oscillating ventilation have obtained inconclusive results [18–21]. The effect of protective ventilation strategies on mortality seems to be related to the decrease in cytokine response induced by mechanical ventilation, minimizing the degree of ventilation-induced lung injury and subsequent biotrauma [22]. The possibilities of nutritional support have also been investigated in depth: recent studies suggest that lipid emulsions and enteral *ω*-3 fatty acid supplementation may have an effect on the inflammatory process, not only in ARDS but in other critical conditions as well.

## 3. *ω*-3 Polyunsaturated Fatty Acids

**Figure 1:**
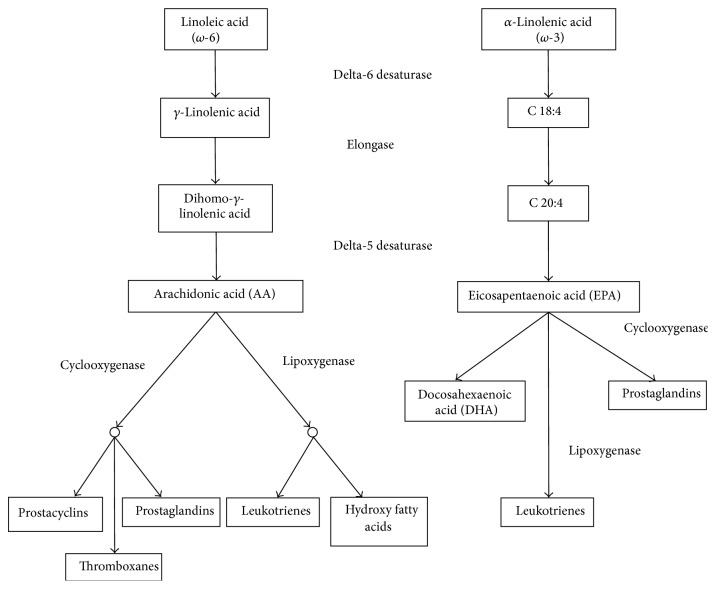
Eicosanoid metabolic pathways.

**Figure 2:**
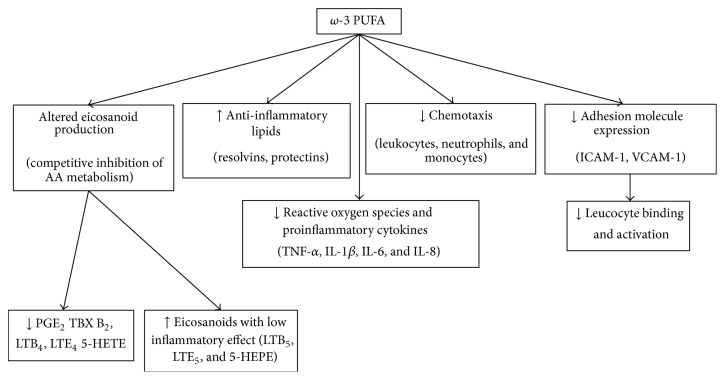
Effects of *ω*-3 polyunsaturated fatty acids in ARDS.

Fatty acids are crucial to human life: they are a main source of energy, they have structural functions as part of the cell membrane, and they participate in cell signalling and response [23]. Essential polyunsaturated fatty acids (PUFA) are linoleic acid (LA, PUFA *ω*-6 series) and linolenic acid (LNA, PUFA *ω*-3 series), which must be obtained through the diet. Linoleic acid is the endogenous precursor of arachidonic acid (AA), which joins the phospholipids in the cell membrane, while LNA is related to eicosapentaenoic acid (EPA) and docosahexaenoic acid (DHA). However, direct ingestion of these acids (from fish, shellfish, and algae) seems to be a more efficient source. Oxygenation and lipoxygenase enzymes transform AA into eicosanoids such as prostaglandins, thromboxanes, and leukotrienes, which have a high biological activity and play a major role in the inflammatory response (Figure 1). In contrast, LNA derivatives are much less active. As they both use the same metabolic routes, *ω*-3 fatty acids compete with AA for the conversion to lipid mediators, balancing the negative effects of *ω*-6 fatty acids [24] (Figure 2).

The prognostic value of eicosanoids in patients with ARDS was studied by Masclans et al. [25] in a prospective study including 21 patients. Plasma levels of thromboxane B2 (TXB2), prostaglandin F1-alpha (PGF1-alpha), and leukotriene B4 (LTB4) were measured both in peripheral and pulmonary arterial samples and in venous samples within 48 h of ARDS onset. ARDS patients showed significantly higher levels of eicosanoids than a reference group of healthy subjects. However, only LTB4 correlated with lung injury score (in peripheral and pulmonary blood). Nonsurvivors presented a lower systemic-pulmonary arterial gradient of eicosanoid levels than survivors. A more recent study assessed the value of LTB4-levels as clinical markers for predicting pulmonary complications such as ARDS, respiratory failure, pulmonary embolism, or pneumonia in multiply traumatized patients [26]. The results showed that LTB4 levels were significantly higher in patients who presented pulmonary complications, suggesting that this mediator may play a role in their pathogenesis.

The possible prognostic value of leukotriene B(4) in ARDS was again reported by Masclans et al. in a prospective study of 16 ARDS patients admitted to the ICU [27]. These authors found higher plasma concentrations of thromboxane B(2), 6-keto-prostaglandin F(1alpha), and leukotriene B(4) compared to the general population, but only leukotriene B(4) was higher in arterial plasma than in mixed venous plasma. Baseline P_a_O_2_/F_I_O_2_ correlated with levels of arterial thromboxane B(2) and arterial leukotriene B(4) and the transpulmonary gradient of leukotriene B(4). A correlation between the transpulmonary gradient of leukotriene B(4) and the lung injury score was also found. Moreover, among nonsurvivors a substantial positive gradient of leukotriene B(4) was detected. Thus, leukotriene B(4) may correlate with lung injury severity and outcome in patients with ARDS.

These results suggest that modulating the inflammatory response through the use of PUFA may be a useful strategy for ARDS treatment.

Classically, the nutrition of critically ill patients was particularly rich in long-chain triglycerides such as linoleic acid. LA may have a harmful effect on the immune and pulmonary function due to its proinflammatory properties, as it is the precursor of AA and eicosanoids. For this reason, in recent years several strategies have been developed in order to minimize these effects: for example, emulsions with a mixture of long-chain and medium-chain fatty acids, enriched with olive oil or with *ω*-3 fatty acids [28]. To date, however, the results regarding the administration of mixed emulsions are inconclusive and the use of olive oil in patients with ARDS has not been studied in depth.

As for the most recent *ω*-3 fatty acids enriched formulas, they may have a beneficial effect on ARDS as they compete with *ω*-6 PUFA and minimize the synthesis of proinflammatory eicosanoids. They contribute to the modulation of nuclear receptor activation (i.e., NF-kappaB suppression), the suppression of arachidonic acid-cyclooxygenase-derived eicosanoids (primarily prostaglandin E(2)) and the alteration of the plasma membrane microorganization related to the function of Toll-like receptors (TLRs), and immune cell recruitment [29].

## 4. Recent Findings in the Use of *ω*-3 PUFA in Patients with ARDS

### 4.1. Experimental Studies

Several experimental studies have reported the effect of *ω*-3 PUFA in animal models. In a murine model, Mancuso et al. [30] compared the effects of fish oil, fish and borage oil, and corn oil. Fish oil and fish and borage oil seemed to improve endotoxin-induced acute lung injury by suppressing the levels of proinflammatory eicosanoids in bronchoalveolar lavage fluid and reducing neutrophil accumulation in lungs. Palombo et al. [31] studied the effect of short-term enteral feeding with eicosapentaenoic acid-enriched or eicosapentaenoic with gamma-linolenic acid-enriched diets in rats. These diets seemed to modulate the fatty acid composition of alveolar macrophage phospholipids, minimizing the formation of less inflammatory eicosanoids.

### 4.2. Human Studies

Research on patients with ARDS dates back to some 15 years (Table 2). Initially, the focus was mainly on the use of a mixture of long-chain and medium-chain fatty acids.

Masclans et al. [32] conducted a randomized trial to evaluate the effect on gas exchange and pulmonary haemodynamics of two different intravenous fat emulsions in 21 patients with ARDS. Patients were randomized to receive a long-chain triglyceride-enriched emulsion (20% LCT), a medium-chain triglyceride/long-chain triglyceride emulsion (20% MCT/LCT: 50/50), or placebo, at a slow rate (2 mg/Kg/min). Increases in cardiac output, oxygen consumption, and oxygen delivery were found during LCT infusion. However, no differences in pulmonary haemodynamics and arterial oxygen tension were detected, suggesting that, at a slow rate, the beneficial effect on cardiac output of LCT infusion offsets the detrimental effect of increased oxygen consumption. No changes were observed in the MCT/LCT group. In this case, the effect of *ω*-3 was not analysed.

Suchner et al. [33] studied the effect of fat emulsions on pulmonary haemodynamics and gas exchange in patients with ARDS or sepsis. In a prospective crossover study, eight patients with ARDS and 10 patients with sepsis were randomized to receive intravenous fat emulsions (LCT/MCT) over 6 h (rapid fat infusion) or 24 h (slow fat infusion) along with a routine parenteral nutrition regimen. In the ARDS group, patients who received rapid fat infusion presented increased prostaglandin I2/thromboxane A2 (P/T) ratios, higher pulmonary shunt, and a decrease in oxygenation. As for the haemodynamics, patients presented decreased pulmonary and systemic vascular resistances while their cardiac indices increased. Increasing plasma concentrations of TxA2 were associated with improved respiratory performance. All values returned to baseline after 12 h. It was therefore suggested that fat emulsion-derived vasodilatory PGI2 may increase pulmonary shunt and affect gas exchange by increasing pulmonary blood flow and decreasing pulmonary vascular tone. The conclusion is that the speed of infusion also plays an important role.

In contrast, patients with severe sepsis showed reduced pulmonary shunt and increased oxygenation index, while the P/T ratio and haemodynamics remained unchanged.

Faucher et al. [34] conducted a crossover study including 18 patients with ARDS who were randomized to receive either a 6 h infusion of a fat emulsion containing LCT or an infusion of 50% LCT/50% MCT. LCT emulsion showed no effect on oxygenation, whereas MCT/LCT emulsion significantly improved P_a_O_2_/F_I_O_2_ by 16% 1 h after initiating the infusion and cardiac output as well. However, the changes were transitory and oxygenation and haemodynamic parameters were similar to baseline at the end of the infusion.

The results regarding the administration of mixed emulsions (LCT/MCT) were not conclusive, probably because the administration of LCT decreases but the ratio *ω*-6/*ω*-3 remains unchanged. Subsequent research focused on the use of *ω*-3 enriched nutrition and obtained promising results. A meta-analysis of three trials including 296 patients published by Pontes-Arruda et al. [35] suggested that enteral supplementation with *ω*-3 fatty acids and gamma-linolenic acid (GLA) could significantly reduce the risk of mortality, the duration of mechanical ventilation, and ICU stay in patients with ARDS.

In 1999, Gadek et al. [36] studied the effect of enteral feeding with eicosapentaenoic acid, gamma-linolenic acid, and antioxidants in patients with ARDS. One hundred and forty-six patients with ARDS were randomized to receive either eicosapentaenoic acid (EPA) + gamma-linolenic acid (GLA) or isonitrogenous, isocaloric standard diet for 4–7 days. Significant improvements in oxygenation were found in patients fed with EPA + GLA compared with controls. These patients also required significantly fewer days of mechanical ventilation and a shorter length of stay in the intensive care unit.

Sabater et al. studied the effect of *ω*-3 fatty acid-enriched lipid emulsion on haemodynamics and gas exchange in patients with ARDS. In a randomized, parallel group study [37], 16 patients were included and randomized to receive the control emulsion (100% LCT) or the study emulsion (50% MCT, 40% LCT, 10% *ω*-3) for 12 h. No differences in gas exchange or haemodynamics were found, except for pulmonary capillary pressure, which was lower in the group with the study emulsion; these results suggested that both emulsions were clinically safe.

In those patients, significant short-term changes in eicosanoid synthesis have been identified [38]. Levels of LTB4, TXB2, and 6-keto-prostaglandin F1-alpha in arterial and mixed venous blood samples increased during infusion in the LCT group and returned to baseline after discontinuation. In the study group, mediator levels decreased during infusion and then behaved erratically. It has been suggested that leukotrienes, particularly LTB4, play a crucial role in lung injury, and their activity may help to modulate the immune response.

Singer et al. [39] reported a beneficial effect of an enteral diet enriched with eicosapentaenoic acid (EPA) and gamma-linolenic acid (GLA) in oxygenation and lung dynamics in 95 patients with ARDS. Those authors found significant differences in P_a_O_2_/F_I_O_2_, but this improvement was lost by day 14. Improvements in static compliance and length of mechanical ventilation were also observed, although these differences were not clinically relevant. No changes in mortality were reported.

However, the characteristics of the control diet must also be taken into consideration. In some of these studies (i.e., Gadek et al., Singer et al.) the control group received a diet with a high content of fatty acids, particularly linoleic acid, which may have had a deleterious effect. What is more, in addition to *ω*-3 FA, formulas also included vitamins, antioxidants, and other elements that could have an effect on the inflammatory reaction, making it difficult to establish the real benefit of each single component on its own. These studies also have some other limitations: for instance, no information was provided on other therapeutic strategies such as mechanical ventilation or fluid administration, which we now know that they have a major role in the management of ARDS. The design of later studies attempted to overcome these limitations.

Studying the effect of enteral nutrition with EPA/GLA and antioxidants in patients in early stages of sepsis without associated organ dysfunction, Pontes-Arruda et al. [40] showed a more frequent progression to severe sepsis and septic shock in the control group, particularly due to development of cardiovascular and respiratory failure. Enteral nutrition with EPA/GLA may then play a beneficial role by slowing the progression of severe forms of sepsis. The study group showed shorter length of stay, lower incidence, and shorter duration of mechanical ventilation, but no differences were found in 28-day all-cause mortality.

The OMEGA study was a randomized multicentre trial which lasted one year and included 272 patients with ARDS [41]. Patients received a twice daily supplementation of *ω*-3 fatty acids, GLA, and antioxidants or an isocaloric control (enteral nutrition was delivered separately). Surprisingly, the study had to be stopped for futility. The enteral diet was reported to be less well tolerated than in previous studies, with a higher incidence of diarrhoea. Patients in the *ω*-3 group had fewer ventilator-free days, ICU-free days, and nonpulmonary organ failure-free days. The authors therefore concluded that enteral supplementation with *ω*-3 fatty acids, GLA, and antioxidants did not improve outcomes and might be harmful. In spite of its early termination this study presented certain methodological advantages over previous trials: mechanical ventilation and fluid administration were controlled in both groups, and the control group received a less proinflammatory diet, as well as a high intake of proteins, which may have had a beneficial effect.

In a phase II multicentre, randomized placebo-controlled trial, Stapleton et al. [42] reported that mechanically ventilated patients with acute lung injury receiving enteral fish oil (EPA and DHA) had increased levels of EPA in BALF with no differences in other biomarkers (e.g., IL-8), organ failure score, ventilator-free days, ICU-free days, or 60-day mortality. Those authors suggested that the positive results of previous studies may have been related to the deleterious effect of control diets. Therefore, the beneficial effect could not be attributed to *ω*-3 PUFA but possibly to other components of the diet or to their combined action.

Grau-Carmona et al. [43] conducted a randomized, open-label study in 11 Spanish intensive care units, including 132 patients with sepsis and established ARDS [31]. Patients were randomized to receive an enteral diet enriched with eicosapentaenoic acid (EPA), gamma-linolenic acid (GLA), and antioxidants or a control diet. No differences in gas exchange or novel organ failures were observed between the two groups.

In contrast, another multicentre randomized trial conducted by Elamin et al. [44] showed an association between an EPA- and GLA-supplemented diet and improvement in gas exchange. Patients in the study group presented reductions in lung injury score, ICU length of stay, and 28-day multiorgan dysfunction score. No differences in survival were detected. However, the sample comprised only 17 patients and so the results should be considered with caution.

In view of the contradictory nature of these results, a systematic review and meta-analysis were recently performed to investigate the beneficial or harmful effect of enteral supplementation of *ω*-3 fatty acids in patients with ARDS [45]. The meta-analysis of seven trials including 955 patients showed no significant decrease in all-cause 28-day mortality or ICU-free days with the use of enteral *ω*-3 fatty acids. Obviously, the analysis had its limitations: most of the trials were performed in small samples and used different control formulas. Nevertheless, it was significant that the *ω*-3 fatty acid-enriched enteral diet was generally well tolerated, with no reports of adverse events.

Nearly all studies considering the effect of *ω*-3 have been conducted using the enteral route. It should be borne in mind that patients with ARDS may present digestive intolerance, due to deep sedation, neuromuscular blockage, or mechanical ventilation, which may limit the use of enteral nutrition. Little is known about the parenteral route in this particular group of patients. This route is worth exploring, as it offers several potential advantages such as the rapid incorporation of the FA into the cell membrane and the possibility of administering higher doses of these lipids.

## 5. Conclusion

The effect of *ω*-3 fatty acid-enriched lipid emulsion or enteral diets on respiratory and cardiovascular variables in patients with ARDS remains unclear. These diets appear to interfere in eicosanoid synthesis, modulating the inflammation response in patients with lung injury. Although enteral *ω*-3 PUFA-enriched diets have a robust physiopathological basis and some promising results were initially reported in experimental and human studies, recent research has cast doubt on their real impact on patients with ARDS. In fact, scientific societies such as the Canadian Society for Clinical Nutrition are currently lowering the level of evidence of these strategies.

The interpretation of the studies is limited by several factors, principally their heterogeneity and methodology. For example, most of the trials are single-centre studies with small sample sizes and include heterogeneous groups of patients with different aetiologies and degrees of severity. Many of them lack information concerning clinical management, such as mechanical ventilation strategies, and different formulas, route of administration, rate of infusion, and treatment duration have been used. The ideal doses, route, and time of administration are still to be established. In general, the effects on oxygenation are transitory and no clear differences in important clinical outcomes such as mortality have been reported. However, the use of lipid formulas seems to be safe and well tolerated.

Based on this evidence, the routine use of *ω*-3 fatty acid-enriched nutrition cannot be recommended. Further large, homogeneous, and high-quality clinical trials need to be conducted to conclusively determine its effectiveness.

## Figures and Tables

**Table 1:** Berlin definition of ARDS.

Timing	Within one week of a known clinical insult or new or worsening respiratory symptoms.

Chest imaging	Bilateral opacities not fully explained by effusions, lobar/lung collapse, or nodules.

Origin of oedema	Respiratory failure not fully explained by cardiac failure or fluid overload. Need for objective assessment (e.g., echocardiography) to exclude hydrostatic oedema if no risk factors are present.

Oxygenation	
Mild	200 mmHg < P_a_O_2_/F_I_O_2_ ≤ 300 mmHg with PEEP or CPAP ≥ 5 cmH_2_O
Moderate	100 mmHg < P_a_O_2_/F_I_O_2_ ≤ 200 mmHg with PEEP ≥ 5 cmH_2_O
Severe	P_a_O_2_/F_I_O_2_ ≤ 100 mmHg with PEEP ≥ 5 cmH_2_O

P_a_O_2_: partial pressure of arterial oxygen; F_I_O_2_: fraction of inspired oxygen; PEEP: positive end expiratory pressure; CPAP: continuous positive airway pressure.

**Table 2:** Omega-3 polyunsaturated fatty acids in ARDS.

Author/year	Design	*N*	Intervention	Main outcomes
Masclans et al., 1998 [32]	RCT, single-centre	21	LCT/MCT (versus LCT versus placebo), 12 hr	↑CO, O_2_ consumption and delivery = Pulmonary haemodynamics and arterial O_2_ tension

Suchner et al., 2001 [33]	RCT (crossover)	18	Rapid (6 hr) versus slow (24 hr) fat emulsion, LCT/MCT	Rapid: ↑P/T, pulmonary shunt and CO; ↓PVR, SVR, and P_a_O_2_/F_I_O_2_

Faucher et al., 2003 [34]	RCT (crossover)	18	LCT/MCT (versus LCT), 6 hr	Transitory ↑P_a_O_2_/F_I_O_2_

Gadek et al., 1999 [36]	RCT, multicentre	146	EPA + GLA, 4–7 days	↑P_a_O_2_/F_I_O_2_ ↓Days of MV and UCI LOS

Sabater et al., 2008 [37]	RCT, single-centre	16	LCT/MCT/*ω*-3 (versus LCT), 12 hr	= Oxygenation, haemodynamics

Sabater et al., 2011 [38]	RCT, single-centre	16	LCT/MCT/*ω*-3 (versus LCT), 12 hr	↓LTB4, TXB2, 6-keto-PG during infusion

Singer et al., 2006 [39]	RCT, single-centre	100	EPA + GLA (versus standard), 14 days	Transitory ↑P_a_O_2_/F_I_O_2_ = Mortality

Pontes-Arruda et al., 2011 [40]	RCT, multicentre	115	EPA + GLA (versus standard), 7 days	↓Severe sepsis and SS ↓Cardiac and respiratory failure ↓Days of MV and UCI LOS = 28-day mortality

Rice et al., 2011 [41]	RCT, multicentre	272	EPA + GLA supplementation (versus standard) twice daily	↓MV-free days, ICU-free days, nonpulmonary organ failure-free days Stopped for futility

Stapleton et al., 2011 [42]	RCT, multicentre	90	EPA + DHA (versus placebo), 14 days	↑Serum EPA = IL-8 in BALF, organ failure score, MV-free days, ICU-free days, and 60-day mortality

Grau-Carmona et al., 2011 [43]	RCT, multicentre	132	EPA + GLA (versus standard)	= Oxygenation and organ failures

Elamin et al., 2012 [44]	RCT, multicentre	17	EPA + GLA (versus standard), 7 days	↓LIS, ICU LOS, 28-day multiorgan dysfunction score = Mortality

RCT: randomized controlled trial; LCT: long-chain triglycerides; MCT: medium-chain triglycerides; CO: cardiac output; EPA: eicosapentaenoic acid; GLA: gamma-linolenic acid; P_a_O_2_/F_I_O_2_: partial pressure of arterial oxygen/fraction of inspired oxygen; MV: mechanical ventilation; ICU LOS: intensive care unit length of stay; P/T: prostaglandin I2/thromboxane A2 ratio; PVR: pulmonary vascular resistance; SVR: systemic vascular resistance; LTB4: leukotriene B4; TXB2: thromboxane B2; 6-keto-PG: 6-keto-prostaglandin; IL-8: interleukin 8; BALF: bronchoalveolar lavage fluid; LIS: lung injury score.
